# Supplementary material for: Prefrontal control of superior colliculus modulates innate escape behavior following adversity
Source: Nat Commun. 2024 Mar 9;15:2158. doi: 10.1038/s41467-024-46460-z (PMC10925020; doi:10.1038/s41467-024-46460-z)
Supplement: Supplementary file 1 — Supplementary Information [file 41467_2024_46460_MOESM1_ESM.pdf]

Supplementary Information:

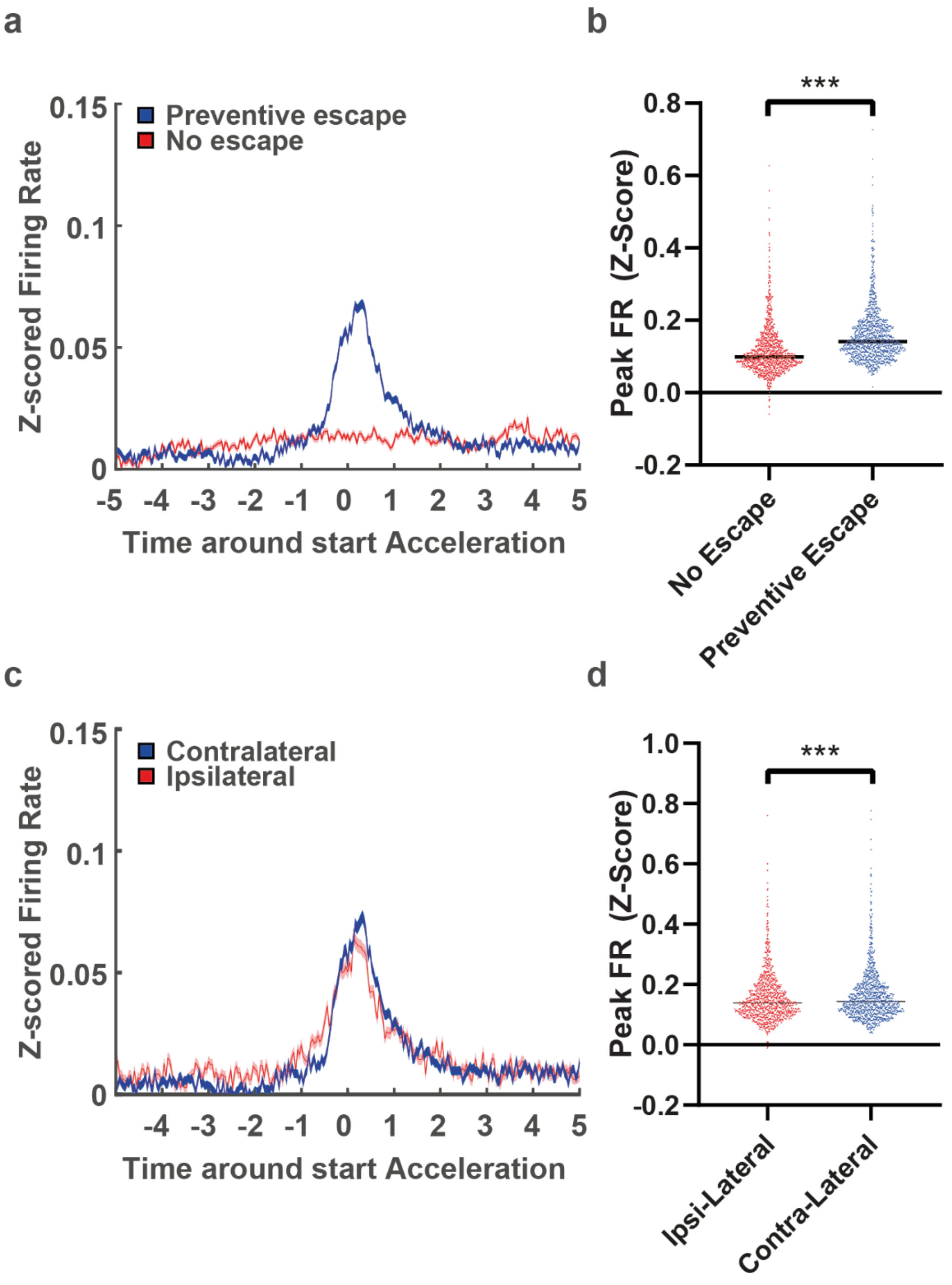

Supplementary figure 1. PE neurons requires escape for response yet are sensitive to the visual lateral bias. (a) Averaged neural response of PE neurons to an incoming robo-beetle when followed by escape (blue) and when no escape follows (red). Each line represents the overall average of the corresponding measure with shaded ±SEM. **(b)** Within unit comparison of peak response with or without escape showing significantly higher response with escape. Within subject ttest ($n = 863$; $p < 0.0001$). **(c)** Averaged

neural response of PE neurons to an incoming robo-beetle coming from the ipsilateral side to the recorded SC (red) and contralateral side (blue). Each line represents the overall average of the corresponding measure with shaded ±SEM. **(d)** Within unit comparison of peak response to ipsi- or contra-lateral threat showing significantly higher response when the beetle arrives from the side contralateral to the recorded SC. Within subject ttest ($n = 840$; $p < 0.0001$). Asterisks indicate significant comparisons (*, $p<.05$; **, $p<.01$; ***, $p<.001$). Source data are provided as a Source Data file.

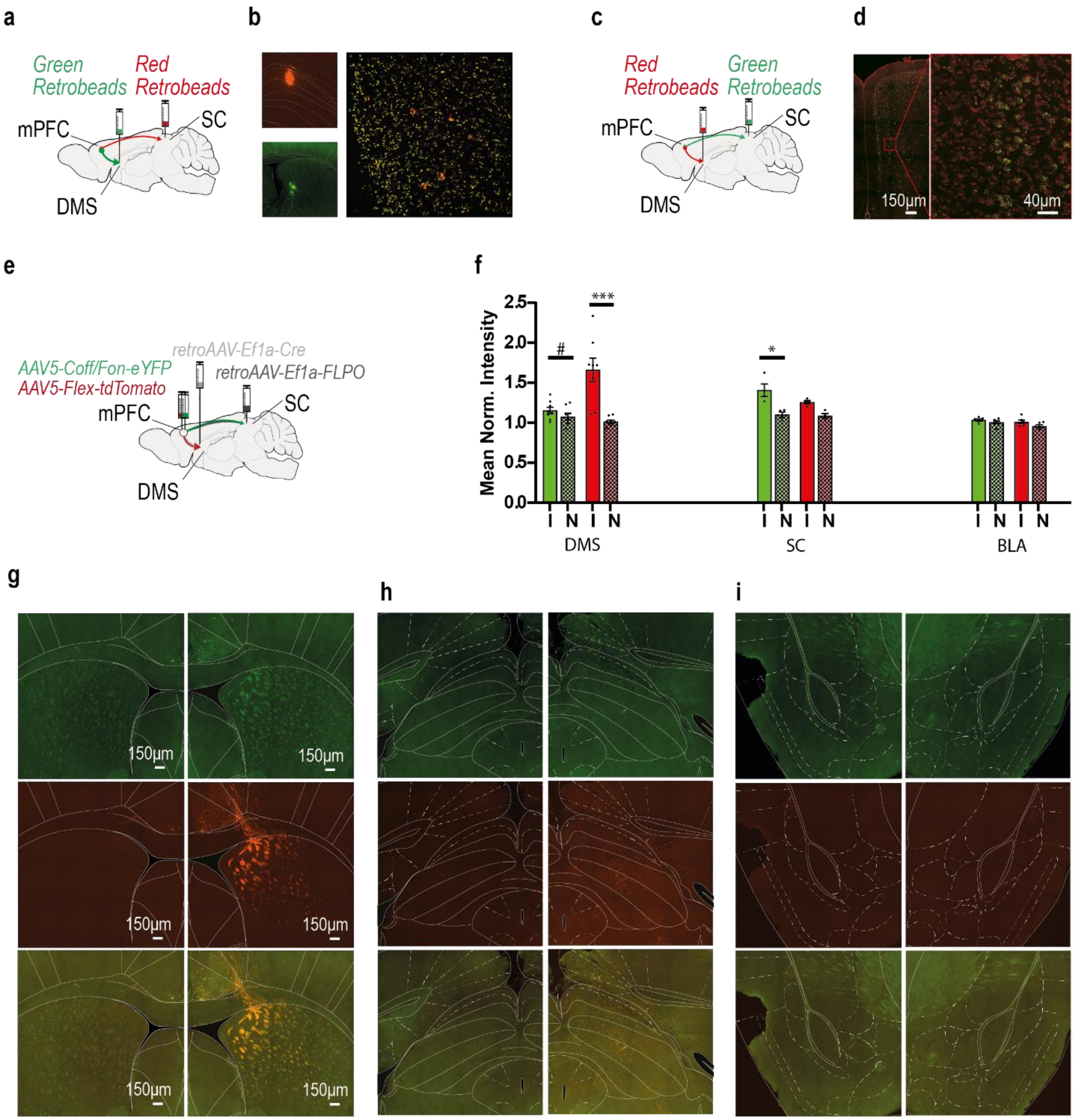

Supplementary figure 2. DMS and SC projecting mPFC neurons overlapping and does not project to the amygdala. (a) A schematic depiction of Retrobeads injection sites at the DMS (green) and the SC (red). **(b)** Representative coronal sections showing red beads at the SC injection site (top left), green beads at the DMS injection site (bottom left) and beads expression including overlap at the mPFC (right). **(c)** A schematic depiction of Retrobeads injection sites at the DMS (red) and the SC (green). **(d)** Representative coronal sections showing beads expression including overlap at the mPFC (right), indicating that these neurons project to both the DMS and the SC. **(e)** A schematic depiction of retro viruses with recombinases injection sites at the DMS and the

SC and the different recombinase dependent fluorophores at the mPFC. **(f)** Comparison of the expression of the different fluorophores at the different projection sites between injected and non-injected sides showing increased expression at the DMS and SC but not in the amygdala. DMS: $n = 8$, SC: $n = 4$, amygdala: $n=6$; SiteXFluorophoreXinjected Three Way ANOVA followed by LSD. DMS-injected-EGFP vs. DMS-non-injected-EGFP $p = 0.08$. DMS-injected-Td-tomato vs. DMS-non-injected-Td-tomato $p < 0.0001$. SC-injected-EGFP vs. SC-non-injected-EGFP $p = 0.0097$. SC-injected-Td-tomato vs. SC-non-injected-Td-tomato $p = 0.14$. amygdala-injected-EGFP vs. amygdala-non-injected-EGFP $p = 0.76$. amygdala-injected-Td-tomato vs. amygdala-non-injected-Td-tomato $p=0.6$. **(g-i)** Example coronal sections of green (top), red (middle) and both (bottom) int injected (left) and non-injected sides of the DMS, SC and amygdala (respectively). In each plot, bars represent means of corresponding measures with error bars showing ±SEM. Asterisks indicate significant post-hoc comparisons (*, $p<.05$; **, $p<.01$; ***, $p<.001$) Pound signs indicate non-significant trends (#, $p<0.1$ - $p>0.05$). Source data are provided as a Source Data file.

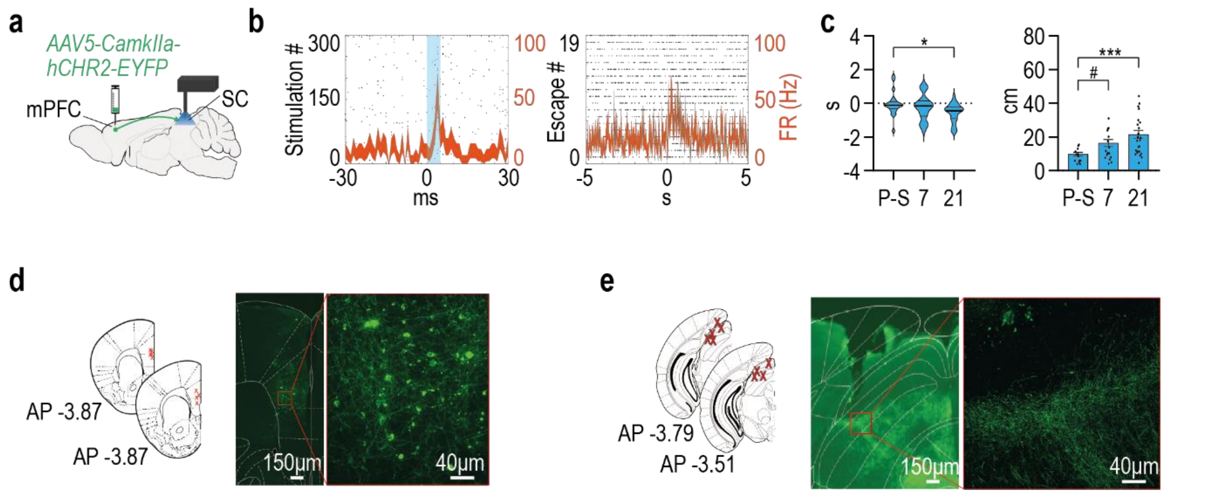

Supplementary figure 3. SC neurons affected directly by general mPFC input, mildly change the neural response after the shock phase. (a) Schematic depiction of optrode placement (SC) and viral injection site(mPFC). **(b)** A raster plot overlaid with a PSTH showing a typical response to light stimulation of mPFC terminals in the SC (left) and PE instances (right) of non-specific mPFC-responsive afferents at the SC. **(c) Left -** In PE-responsive units, spike initiation times (ms) relative to escape onset seem to undergo a shift afore in distribution (n = 21 pre-shock; n=24 on 7th day; n=23 on the 21st day), Kruskal–Wallis H (2) = 7.645 $p < 0.05$ Dunn's multiple comparisons Pre-shock vs. 7th day $p = 0.660$; Pre-shock vs. 21st day $p < 0.05$. **Right** - spike initiation occurs at a greater distance (cm) from beetle 21 days following footshock (n = 14 pre-shock; n=17 on 7th day; n=24 on the 21st day). One way ANOVA with Dunnet's Pre-shock vs. 7th day $p = 0.082$; Pre-shock vs. 21st day $p < 0.01$. **(d)** Histologically confirmed injection sites at the mPFC, marked by X's, from all included animals. Right: representative coronal section showing viral expression in mPFC projections cells. **(e)** Histologically confirmed optrode placement sites at the mPFC, marked by X's, from all included animals. Right: representative coronal section showing viral expression of mPFC projections in the SC.
In each plot, bars represent means of corresponding measures with error bars showing ±SEM. In violin graphs, the bold midlines represent medians with IQR in regular lines above and below them. In peri-event histograms, each line is representing the overall average of the corresponding measure with shaded ±SEM. Asterisks indicate significant post-hoc comparisons (*, p<0.05; ***, p<0.001). Pound signs indicate non-significant trends (#, p<0.1 - p>0.05). Source data are provided as a Source Data file.

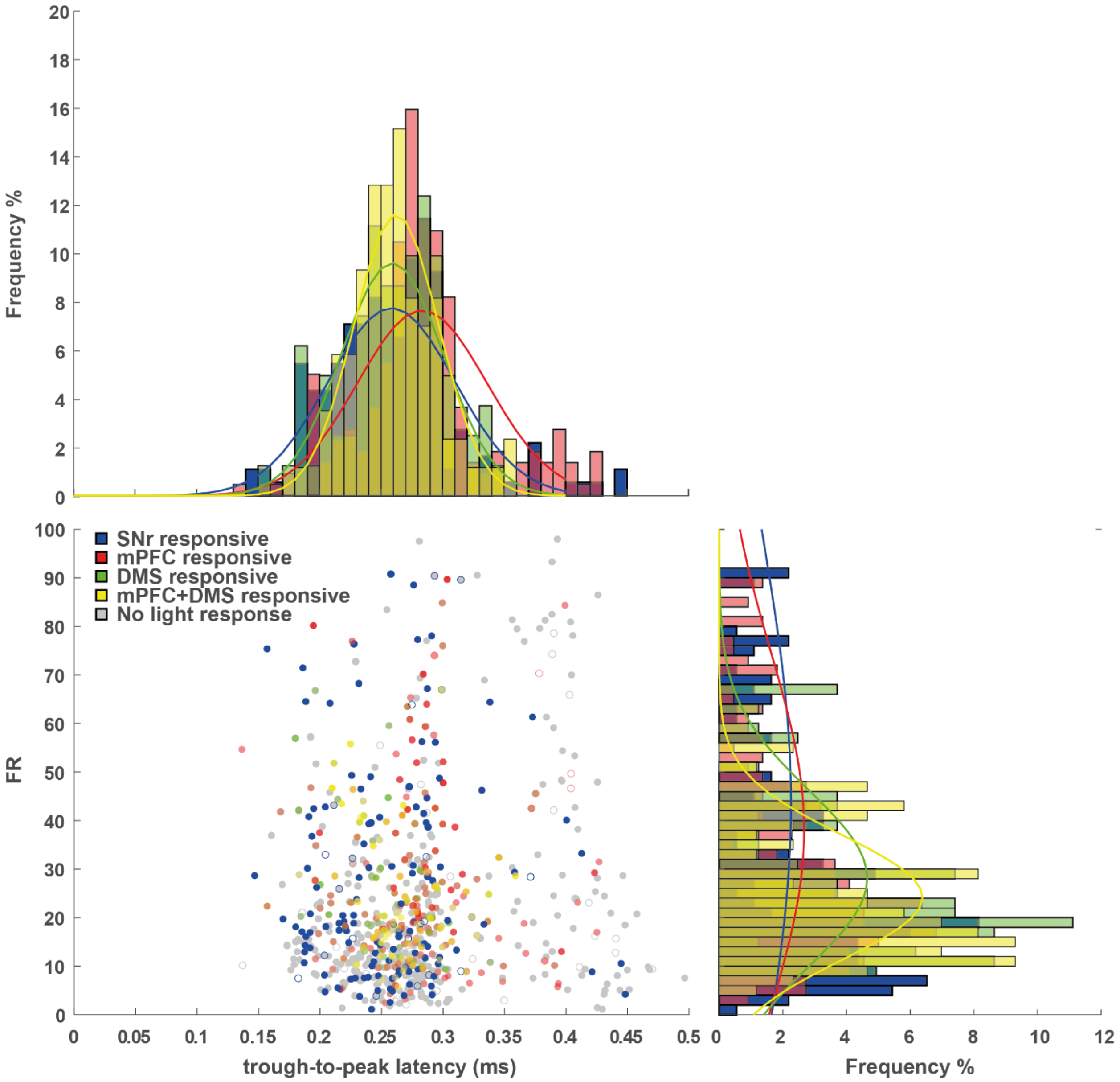

Supplementary figure 4. Characterizing SC recorded units on a two dimensional space. X-axis represents trough-to-peak latency, while Y-axis represents the firing rate of the unit. Each color represents a different cohort of units according to their photo-tag response. Each line represents the overall average of the corresponding measure with shaded ±SEM. The different SC neuron groups, by response to different afferents, mPFC and DMS (n=86), mPFC (n=220), DMS (n=81), SNr (n=184) differ in trough to peak latency. One way ANOVA with Tukey's mPFC-DMS vs mPFC $p<0.05$; mPFC-DMS vs DMS $p=0.972$; mPFC-DMS vs nr $p=0.931$; mPFC vs DMS $p<0.001$; mPFC vs SNr $p<0.0001$; DMS vs SNr $p=0.9998$. The different SC neuron groups, by response to

different afferents also differ in their firing rates. One way ANOVA with Tukey's mPFC-DMS vs mPFC $p<0.05$; mPFC-DMS vs DMS $p=0.919$; mPFC-DMS vs nr $p<0.001$; mPFC vs DMS $p=0.221$; mPFC vs SNr $p=0.486$; DMS vs SNr $p<0.05$. Source data are provided as a Source Data file.

Supplementary tables:

Statistics table:

Figure	Group	N	Mean	SD
1d	Shock group - Pre-Shock	19	28.05263	1.535
	Shock group - Post 7 Days	19	39.57895	2.394
	Shock group - Post 21 Days	19	42.52632	2.932
	No-Shock group - Pre-Shock	13	22.23077	1.856
	No-Shock group - Post 7 Days	13	16.46154	2.895
	No-Shock group - Post 21 Days	13	18.69231	3.544
	**Effect**	**Test**	**F (DFn, DFd)**	**p**
	Main Effect Group	Mixed- two way ANOVA	F (1,30) = 55.94	<0.0001
	Time X Group interaction	Mixed- two way ANOVA	F (2,60) = 8.5	<0.001
	Shock Pre-Shock vs. 7 Days	Tukey post-hoc comparinsons		<0.01
	Shock Pre-Shock vs. 21 Days	Tukey post-hoc comparinsons		<0.001
	No-Shock Pre-Shock vs. 7 Days	Tukey post-hoc comparinsons		0.657
	No-Shock Pre-Shock vs. 21 Days	Tukey post-hoc comparinsons		0.938
	Shock vs No-Shock PS	Tukey post-hoc comparinsons		0.618
	Shock vs No-Shock 7 Days	Tukey post-hoc comparinsons		<0.001
	Shock vs No-Shock 21 Days	Tukey post-hoc comparinsons		<0.001

Figure	Group	N	Mean	SD
1e	Shock group - Pre-Shock	19	5.451462	0.344
	Shock group - Post 7 Days	19	6.377145	0.329
	Shock group - Post 21 Days	19	6.394457	0.293
	No-Shock group - Pre-Shock	13	5.983556	0.416
	No-Shock group - Post 7 Days	13	5.324654	0.398
	No-Shock group - Post 21 Days	13	5.855558	0.354
	**Effect**	**Test**	**F (DFn, DFd)**	**p**

	Time X Group interaction	Mixed- two way ANOVA	F (2,60) = 2.558	0.085
	Shock Pre-Shock vs. 7 Days	Tukey post-hoc comparinsons		<0.05
	Shock Pre-Shock vs. 21 Days	Tukey post-hoc comparinsons		<0.05
	No-Shock Pre-Shock vs. 7 Days	Tukey post-hoc comparinsons		0.236
	No-Shock Pre-Shock vs. 21 Days	Tukey post-hoc comparinsons		0.817
	Shock vs No-Shock PS	Tukey post-hoc comparinsons		0.296
	Shock vs No-Shock 7 Days	Tukey post-hoc comparinsons		<0.05
	Shock vs No-Shock 21 Days	Tukey post-hoc comparinsons		0.306

Figure	Group	N	Mean	SD
1f	Shock group - Pre-Shock	19	19.92085	3.128
	Shock group - Post 7 Days	19	9.43582	1.901
	Shock group - Post 21 Days	19	10.76216	1.910
	No-Shock group - Pre-Shock	13	18.8639	3.781
	No-Shock group - Post 7 Days	13	17.17114	2.298
	No-Shock group - Post 21 Days	13	18.28754	2.309
	Effect	Test	F (DFn, DFd)	p
	Main Effect Group	Mixed- two way ANOVA	F (1,30) = 3.07	0.089
	Main Effect Time	Mixed- two way ANOVA	F (2,60) = 4.14	<0.05
	Time X Group interaction	Mixed- two way ANOVA	F (2,60) = 2.51	0.089
	Shock Pre-Shock vs. 7 Days	Tukey post-hoc comparinsons		<0.0001
	Shock Pre-Shock vs. 21 Days	Tukey post-hoc comparinsons		<0.001
	No-Shock Pre-Shock vs. 7 Days	Tukey post-hoc comparinsons		0.626
	No-Shock Pre-Shock vs. 21 Days	Tukey post-hoc comparinsons		0.868

	Shock vs No-Shock PS	Tukey post-hoc comparinsons		0.778
	Shock vs No-Shock 7 Days	Tukey post-hoc comparinsons		<0.05
	Shock vs No-Shock 21 Days	Tukey post-hoc comparinsons		<0.05

Figure	Group	N	Mean	SD
1h	Pre-Shock	16	7.438	0.677
	Post 7 Days	16	11.438	1.288
	Post 21 Days	16	12	1.017
	Effect	Test	F (DFn, DFd)	p
	Main Effect	Repeated one way ANOVA	F (2, 30) = 6.651	<0.05
	Pre-Shock vs. 7 Days	Tukey post-hoc comparinsons		<0.01
	Pre-Shock vs. 21 Days	Tukey post-hoc comparinsons		<0.01

Figure	Group	N	Mean	SD
1i	Pre-Shock	20	4.930	0.153
	Post 7 Days	20	5.756	0.304
	Post 21 Days	20	5.907	0.413
	Effect	Test	F (DFn, DFd)	p
	Main Effect	Repeated one way ANOVA	F (2, 38) = 3.401	<0.05
	Pre-Shock vs. 7 Days	Tukey post-hoc comparinsons		<0.05
	Pre-Shock vs. 21 Days	Tukey post-hoc comparinsons		<0.05

Figure	Group	N	Mean	SD
1k	Pre-Shock	15	7.6	0.696
	Post 7 Days	15	12.066	1.513
	Post 21 Days	15	14.466	1.684
	Effect	Test	F (DFn, DFd)	p
	Main Effect	Repeated one way ANOVA	F (2, 28) = 5.682	<0.01

	Pre-Shock vs. 7 Days	Tukey post-hoc comparinsons		<0.05
	Pre-Shock vs. 21 Days	Tukey post-hoc comparinsons		<0.01

Figure	Group	N	Mean	SD
1l	Pre-Shock	16	6.427	0.439
	Post 7 Days	16	6.694	0.349
	Post 21 Days	16	7.750	0.649
	Effect	Test	F (DFn, DFd)	p
	Main Effect	Repeated one way ANOVA	F (2, 30) = 1.862	0.173

Figure	Group	N	Median	IQR
2e left	Pre-Shock	443	-640	-1280 to -120
	Post 7 Days	362	-440	-1240 to -40
	Post 21 Days	310	-1020	-1840 to -480
	Effect	Test	H (DF)	p
	Main Effect	Kruskal-Wallis test	H (2) = 49.65	<0.0001
	Pre-Shock vs. 7 Days	Dunn's multiple comparisons test		0.067
	Pre-Shock vs. 21 Days	Dunn's multiple comparisons test		<0.0001

Figure	Group	N	Mean	SD
2e right	Pre-Shock	370	28.08	7.303
	Post 7 Days	279	28.01	6.795
	Post 21 Days	283	28.12	6.258
	Effect	Test	F (DFn, DFd)	p
	Main Effect	One-way ANOVA	F (2, 929) = 0.0162	0.984

Figure	Group	N	Median	IQR

	Pre-Shock	333	-200	-640 to 40
	Post 7 Days	272	-280	-640 to 40
	Post 21 Days	259	-480	-920 to -200
	Effect	Test	H (DF)	p
	Main Effect	Kruskal-Wallis test	H (2) = 33.05	<0.0001
2f left	Pre-Shock vs. 7 Days	Dunn's multiple comparisons test		0.736
	Pre-Shock vs. 21 Days	Dunn's multiple comparisons test		<0.0001

Figure	Group	N	Mean	SD
	Pre-Shock	237	15.39	10.610
	Post 7 Days	203	15.47	9.860
	Post 21 Days	220	17.85	9.031
	Effect	Test	F (DFn, DFd)	p
	Main Effect	Mixed-effects model (REML)	F (2, 657) = 4.408	<0.05
2f right	Pre-Shock vs. 7 Days	Dunnett's multiple comparisons test		0.995
	Pre-Shock vs. 21 Days	Dunnett's multiple comparisons test		<0.05

Figure	Group	N	Median	IQR
	Pre-Shock	250	-200	-760 to 80
	Post 7 Days	202	-160	-680 to 160
2g left	Post 21 Days	177	-280	-800 to 60
	Effect	Test	H (DF)	p
	Main Effect	Kruskal-Wallis test	H (2) = 4.464	0.107

Figure	Group	N	Mean	SD
	Pre-Shock	174	14.32	7.343

2g right	Post 7 Days	123	14.4	6.182
	Post 21 Days	128	15.5	5.844
	Effect	Test	F (DFn, DFd)	p
	Main Effect	One-way ANOVA	F (2, 422) = 1.351	0.260

Figure	Group	N	Median	IQR
3f left	Pre-Shock	38	-440	-1090 to -30
	Post 7 Days	19	-200	-480 to 240
	Post 21 Days	16	-360	-790 to 70
	Effect	Test	H (DF)	p
	Main Effect	Kruskal-Wallis test	H (2) = 5.758	0.056

Figure	Group	N	Mean	SD
3f right	Pre-Shock	29	17	10.660
	Post 7 Days	13	15.11	9.114
	Post 21 Days	11	15.13	7.303
	Effect	Test	F (DFn, DFd)	p
	Main Effect	One-way ANOVA	F (2, 50) = 0.247	0.144

Figure	Group	N	Mean	SD
4f	mPFC positive	144	7.847	6.253
	mPFC negative	40	14.988	6.062
	DMS positive	129	17.209	6.450
	DMS negative	37	16.811	7.735
	Effect	Test	F (DFn, DFd)	p
	Intraction	Two-way ANOVA	F (1, 346) = 20.32	<0.0001
	Positive:mPFC vs. Negative:DMS	Tukey's multiple comparisons test		<0.0001
	Positive:mPFC vs. Negative:mPFC	Tukey's multiple comparisons test		<0.0001
	Positive:mPFC vs. Positive:DMS	Tukey's multiple		<0.0001

		comparisons test		
	Negative:mPFC vs. Negative:DMS	Tukey's multiple comparisons test		0.232
	Positive:DMS vs. Negative:DMS	Tukey's multiple comparisons test		0.988
	Positive:DMS vs. Negative:mPFC	Tukey's multiple comparisons test		0.605

Figure	Group	N	Median	IQR
5b top	Pre-Shock	44	-60	-390 to 80
	Post 7 Days	51	-480	-840 to -200
	Post 21 Days	35	-480	-800 to -120
	Effect	Test	H (DF)	p
	Main Effect	Kruskal-Wallis test	H (2) = 22.74	<0.0001
	Pre-Shock vs. 7 Days	Dunn's multiple comparisons test		<0.0001
	Pre-Shock vs. 21 Days	Dunn's multiple comparisons test		<0.001

Figure	Group	N	Mean	SD
5b bottom	Pre-Shock	29	11.53	4.594
	Post 7 Days	47	17.64	10.160
	Post 21 Days	31	17.13	7.552
	Effect	Test	F (DFn, DFd)	p
	Main Effect	One-way ANOVA	F (2, 104) = 5.478	<0.01
	Pre-Shock vs. 7 Days	Dunnett's multiple comparisons test		<0.01

	Pre-Shock vs. 21 Days	Dunnett's multiple comparisons test		<0.05

Figure	Group	N	Median	IQR
5d top	Pre-Shock	58	-200	-520 to 40
	Post 7 Days	56	-340	-900 to -170
	Post 21 Days	52	-420	-800 to -240
	Effect	Test	H (DF)	p
	Main Effect	Kruskal-Wallis test	H (2) = 17.03	<0.001
	Pre-Shock vs. 7 Days	Dunn's multiple comparisons test		<0.01
	Pre-Shock vs. 21 Days	Dunn's multiple comparisons test		<0.001

Figure	Group	N	Mean	SD
5d bottom	Pre-Shock	37	12.44	4.042
	Post 7 Days	52	16.37	9.975
	Post 21 Days	50	16.61	8.416
	Effect	Test	F (DFn, DFd)	p
	Main Effect	One-way ANOVA	F (2, 136) = 3.323	<0.05
	Pre-Shock vs. 7 Days	Dunnett's multiple comparisons test		<0.05
	Pre-Shock vs. 21 Days	Dunnett's multiple comparisons test		<0.05

Figure	Group	N	Mean	SD
6d left	Control Pre-Shock	10	10.600	5.100
	Control 7 Days	10	11.600	5.540

	Control 21 Days	10	11.200	6.160
	Hm4D Pre-Shock	9	10.220	5.520
	Hm4Dl 7 Days	8	10.630	8.140
	Hm4D 21 Days	9	8.440	7.190
	Effect	Test	F (DFn, DFd)	p
	Intraction	Two-way mixed ANOVA	F (2, 33) = 0.389	0.680

Figure	Group	N	Mean	SD
6d right	Control 7 Days	10	142.970	106.730
	Control 21 Days	10	137.370	100.560
	Hm4Dl 7 Days	8	85.890	33.600
	Hm4D 21 Days	9	82.040	45.360
	Effect	Test	F (DFn, DFd)	p
	Main Effect, Group	Two-way ANOVA	F (1, 33) =4.389	<0.05

Figure	Group	N	Mean	SD
6e	Control Pre-Shock	10	4.700	1.350
	Control 7 Days	10	7.220	2.590
	Control 21 Days	10	6.620	2.270
	Hm4D Pre-Shock	9	5.060	1.890
	Hm4Dl 7 Days	10	5.020	1.670
	Hm4D 21 Days	10	5.300	1.500
	Effect	Test	F (DFn, DFd)	p
	Intraction	Two-way mixed ANOVA	F (2, 35) = 3.268	<0.05
	Control P-S vs. 7	Tukey's multiple		<0.01

		comparisons test		
	Control P-S vs. 21	Tukey's multiple comparisons test		<0.05
	Control 7 vs. 21	Tukey's multiple comparisons test		0.666
	Hm4D P-S vs. 7	Tukey's multiple comparisons test		>0.9999
	Hm4D P-S vs. 21	Tukey's multiple comparisons test		0.916
	Hm4D 7 vs. 21	Tukey's multiple comparisons test		0.912

Figure	Group	N	Mean	SD
6f	Control Pre-Shock	10	23.440	11.110
	Control 7 Days	9	13.081	7.259
	Control 21 Days	9	25.647	13.337
	Hm4D Pre-Shock	10	18.358	7.243
	Hm4Dl 7 Days	10	15.702	7.696
	Hm4D 21 Days	10	19.116	10.945
	Effect	Test	F (DFn, DFd)	p
	Intraction	Two-way mixed ANOVA	F (2, 34) = 1.656	0.206
	Main Effect, Time	Two-way mixed ANOVA	F (2, 34) = 5.050	<0.05

	Control P-S vs. 7	Dunn's multiple comparisons test		<0.05
	Control P-S vs. 21	Tukey's multiple comparisons test		0.662
	Hm4D P-S vs. 7	Tukey's multiple comparisons test		0.685
	Hm4D P-S vs. 21	Tukey's multiple comparisons test		0.968

Figure	Group/Phase	N	Mean	SD
7e	Control 60cm	7	0.286	0.488
	Control None	7	4.857	1.864
	Control 10cm	7	5.143	2.410
	Control 20cm	7	4.571	1.988
	ChR2 60cm	8	2.000	2.390
	ChR2 None	8	6.625	2.560
	ChR2 10cm	8	8.375	4.173
	ChR2 20cm	8	7.750	3.151
	Effect	Test	F (DFn, DFd)	p
	Main Effect, Group	Two-way repeated measures ANOVA	F (1, 13) = 6.468	<0.05
	Control vs ChR2: 60cm	Fisher's LSD		0.213

	Control vs ChR2: None	Fisher's LSD		0.199
	Control vs ChR2: 10cm	Fisher's LSD		<0.05
	Control vs ChR2: 20cm	Fisher's LSD		<0.05
	Main Effect, Phase	Two-way repeated measures ANOVA	$F (3, 39) = 22.09$	<0.0001
	60cm vs. None	Tukey's multiple comparisons test		<0.0001
	60cm vs.10cm	Tukey's multiple comparisons test		<0.0001
	60cm vs. 20cm	Tukey's multiple comparisons test		<0.0001
	None vs. 10cm	Tukey's multiple comparisons test		0.559
	None vs. 20cm	Tukey's multiple comparisons test		0.948
	10cm vs. 20cm	Tukey's multiple comparisons test		0.866

Figure	Group/Phase	N	Mean	SD
7f	Control None	7	4.923	2.208
	Control 10cm	7	4.634	1.368
	Control 20cm	7	5.503	1.162

	ChR2 None	8	4.892	1.659
	ChR2 10cm	8	6.307	0.880
	ChR2 20cm	8	8.980	2.742
	Effect	Test	F (DFn, DFd)	p
	Main Effect, Group	Two-way repeated measures ANOVA	F (1, 13) = 13.70	<0.01
	Main Effect, Phase	Two-way repeated measures ANOVA	F (2, 26) = 6.070	<0.01
	None vs. 10cm	Tukey's multiple comparisons test		0.428
	None vs. 20cm	Tukey's multiple comparisons test		<0.01
	10cm vs. 20cm	Tukey's multiple comparisons test		<0.05
	Interaction	Two-way repeated measures ANOVA	F (2, 26) = 3.146	0.060
	Control: None vs. 10cm	Tukey's multiple comparisons test		0.957
	Control: None vs. 20cm	Tukey's multiple comparisons test		0.838
	Control: 10cm vs. 20cm	Tukey's multiple comparisons test		0.676

	ChR2: None vs. 10cm	Tukey's multiple comparisons test		0.316
	ChR2: None vs. 20cm	Tukey's multiple comparisons test		<0.001
	ChR2l: 10cm vs. 20cm	Tukey's multiple comparisons test		<0.05
	Control vs ChR2: None	Fisher's LSD		0.974
	Control vs ChR2: 10cm	Fisher's LSD		0.080
	Control vs ChR2: 20cm	Fisher's LSD		<0.001

Figure	Group	N	Mean	SD
Supp. 1b	PE	864	0.1649	0.07384
	no escape	864	0.1196	0.08135
	Effect	Test	T (DF)	p
	Main Effect	ttest (within subject)	t(863)=17.72	<0.0001

Figure	Group	N	Mean	SD
Supp. 1d	Ipsi	840	0.1597	0.07999
	Contra	840	0.169	0.08951
	Effect	Test	T (DF)	p
	Main Effect	ttest (within subject)	t(840)=4.208	<0.0001

Figure	Region/Side/Fluorophore	N	Mean	SD
Supp 2f	DMS injected EGFP	8	1.155	0.116
	DMS injected Td Tommato	8	1.662	0.412
	DMS non- injected EGFP	8	1.077	0.123
	DMS non injected Td Tommato	8	1.014	0.060
	SC injected EGFP	4	1.407	0.155
	SC injected Td Tommato	4	1.258	0.042
	SC non- injected EGFP	4	1.104	0.062
	SC non injected Td Tommato	4	1.089	0.053

	amygdala injected EGFP	6	1.035	0.031
	amygdal injected Td Tommato	6	1.012	0.054
	amygdal non- injected EGFP	6	1.006	0.046
	amygdal non injected Td Tommato	6	0.957	0.040
	Effect	Test	F (DFn, DFd)	p
	Interaction	Three-way ANOVA	F (2, 60) = 8.251	<0.001
	DMS injected EGFP vs non-injected	Fisher's LSD		p=0.08
	DMS injected Td-Tomato vs non-injected	Fisher's LSD		<0.0001
	SC injected EGFP vs non-injected	Fisher's LSD		<0.01
	SC injected Td-Tomato vs non-injected	Fisher's LSD		p=0.14
	amg injected EGFP vs non-injected	Fisher's LSD		p=0.76
	amg injected Td-Tomato vs non-injected	Fisher's LSD		p=0.60

Figure	Group	N	Median	IQR
Supp. 3c left	Pre-Shock	21	-120	-340 to 100
	Post 7 Days	24	-160	-770 to 130
	Post 21 Days	23	-440	-920 to -240
	Effect	Test	H (DF)	p
	Main Effect	Kruskal-Wallis test	H (2) = 7.645	<0.05
	Pre-Shock vs. 7 Days	Dunn's multiple comparisons test		0.660
	Pre-Shock vs. 21 Days	Dunn's multiple comparisons test		<0.05

Figure	Group	N	Mean	SD
Supp. 3c right	Pre-Shock	14	9.686	3.883
	Post 7 Days	17	16.38	8.560
	Post 21 Days	24	21.47	11.340
	Effect	Test	F (DFn, DFd)	p

	Main Effect	One-way ANOVA	F (2, 52) = 7.416	<0.01
	Pre-Shock vs. 7 Days	Dunnett's multiple comparisons test		0.082
	Pre-Shock vs. 21 Days	Dunnett's multiple comparisons test		<0.01

Figure	Group	N	Mean	SD
Supp. 4 Trough to Peak latency	mPFC-DMS	86	0.2624	0.034
	mPFC	220	0.2835	0.053
	DMS	81	0.2592	0.042
	SNr	184	0.2586	0.052
	Effect	Test	F (DFn, DFd)	p
	Main Effect	One-way ANOVA	F (3, 567) = 10.9258	<0.0001
	mPFC-DMS vs mPFC	Tukey		<0.05
	mPFC-DMS vs DMS	Tukey		0.972
	mPFC-DMS vs SNr	Tukey		0.931
	mPFC vs DMS	Tukey		<0.001
	mPFC vs SNr	Tukey		< 0.0001
	DMS vs SNr	Tukey		P=0.9998

Figure	Group	N	Mean	SD
Supp. 4 FR	mPFC-DMS	86	24.1586	12.815
	mPFC	220	37.9782	36.890
	DMS	81	28.0892	18.153
	SNr	184	43.6155	55.080
	Effect	Test	F (DFn, DFd)	p
	Main Effect	One-way ANOVA	F (3, 567) = 6.0505	<0.0005
	mPFC-DMS vs mPFC	Tukey		<0.05
	mPFC-DMS vs DMS	Tukey		0.919
	mPFC-DMS vs SNr	Tukey		<0.001
	mPFC vs DMS	Tukey		0.221
	mPFC vs SNr	Tukey		0.486
	DMS vs SNr	Tukey		<0.05
