# Supplementary material for: Prefrontal control of superior colliculus modulates innate escape behavior following adversity
Source: Nat Commun. 2024 Mar 9;15:2158. doi: 10.1038/s41467-024-46460-z (PMC10925020; doi:10.1038/s41467-024-46460-z)
Supplement: Supplementary file 3 — Reporting Summary [file 41467_2024_46460_MOESM3_ESM.pdf]

nature portfolio

Corresponding author(s): Oded Klavir

Last updated by author(s): Feb 21, 2024

Reporting Summary

Nature Portfolio wishes to improve the reproducibility of the work that we publish. This form provides structure for consistency and transparency in reporting. For further information on Nature Portfolio policies, see our Editorial Policies and the Editorial Policy Checklist.

Statistics

For all statistical analyses, confirm that the following items are present in the figure legend, table legend, main text, or Methods section.

n/a	Confirmed	
☐	☒	The exact sample size (n) for each experimental group/condition, given as a discrete number and unit of measurement
☐	☒	A statement on whether measurements were taken from distinct samples or whether the same sample was measured repeatedly
☐	☒	The statistical test(s) used AND whether they are one- or two-sided<br>*Only common tests should be described solely by name; describe more complex techniques in the Methods section.*
☒	☐	A description of all covariates tested
☐	☒	A description of any assumptions or corrections, such as tests of normality and adjustment for multiple comparisons
☐	☒	A full description of the statistical parameters including central tendency (e.g. means) or other basic estimates (e.g. regression coefficient) AND variation (e.g. standard deviation) or associated estimates of uncertainty (e.g. confidence intervals)
☐	☒	For null hypothesis testing, the test statistic (e.g. F, t, r) with confidence intervals, effect sizes, degrees of freedom and P value noted<br>*Give P values as exact values whenever suitable.*
☒	☐	For Bayesian analysis, information on the choice of priors and Markov chain Monte Carlo settings
☒	☐	For hierarchical and complex designs, identification of the appropriate level for tests and full reporting of outcomes
☒	☐	Estimates of effect sizes (e.g. Cohen's d, Pearson's r), indicating how they were calculated

Our web collection on statistics for biologists contains articles on many of the points above.

Software and code

Policy information about availability of computer code

Data collection	Extracellular waveform signals were recorded using Digital Lynx hardware and software (Neuralynx Inc; USA), Neural data was sorted manually using Offline Sorter (version 4.6.0; 2021; Plexon; USA) and analyzed using custom codes in Matlab (Version R2021a). All measures regarding the animals' behavior were detected and extracted using DeepLabCut2 (Mathis et al., 2018) and a custom made code in Matlab (Version R2021a) and made available on open science framework (OSF) at: https://osf.io/h7r4c/ . Where online detection of mouse-beetle proximity was needed, Ethovision XT (v14; 2018; Noldus) was used for this task.
Data analysis	Statistical analyses were performed using Prism 8.0 (GraphPad) Statistica 13.05 (TIBCO) and Matlab (Version R2021a).

For manuscripts utilizing custom algorithms or software that are central to the research but not yet described in published literature, software must be made available to editors and reviewers. We strongly encourage code deposition in a community repository (e.g. GitHub). See the Nature Portfolio guidelines for submitting code & software for further information.

Data

Policy information about availability of data

All manuscripts must include a data availability statement. This statement should provide the following information, where applicable:

- Accession codes, unique identifiers, or web links for publicly available datasets
- A description of any restrictions on data availability
- For clinical datasets or third party data, please ensure that the statement adheres to our policy

Some of the data to support this study is available within the paper's figures and supplementary information. All the data is available from the corresponding author upon reasonable request. Source data are also provided with this paper.

Research involving human participants, their data, or biological material

Policy information about studies with human participants or human data. See also policy information about sex, gender (identity/presentation), and sexual orientation and race, ethnicity and racism.

Reporting on sex and gender	N/A
Reporting on race, ethnicity, or other socially relevant groupings	N/A
Population characteristics	N/A
Recruitment	N/A
Ethics oversight	N/A

Note that full information on the approval of the study protocol must also be provided in the manuscript.

Field-specific reporting

Please select the one below that is the best fit for your research. If you are not sure, read the appropriate sections before making your selection.

☒ Life sciences ☐ Behavioural & social sciences ☐ Ecological, evolutionary & environmental sciences

For a reference copy of the document with all sections, see nature.com/documents/nr-reporting-summary-flat.pdf

Life sciences study design

All studies must disclose on these points even when the disclosure is negative.

Sample size	Sample sizes were determined according to measures and sample sizes, previously reported in the literature (Behavior, Electrophysiology and Optogenetics: Courtin et al., 2013, PMID:24256726; Tovote et al., 2016, PMID:27279213; Behavior and Chemogenetics: Vetere et al., 2017, PMID:28426969; Luchicchi et al., 2016, PMID:27630545)
Data exclusions	Mice with incorrect injection or implant sites were excluded from data analysis.<br>Mice with missing values were excluded from repeated measures analyses.
Replication	All behavioral and electrophysiological experiments were replicated at least twice in this study with all replications successful. The behavioral paradigm was also replicated in several previous studies (Heinz et al., 2017, PMID:28890367; Almada et al., 2018, PMID:29867370; Heinz et al., 2021, PMID:34039953) .
Randomization	Animals of the same litter were randomly assigned to experimental groups.
Blinding	Blinding was used in all experiments. The only factor that could not be blinded in elcectrophysiological experiments (figures 2-5) is the side of the recording electrode.

Reporting for specific materials, systems and methods

We require information from authors about some types of materials, experimental systems and methods used in many studies. Here, indicate whether each material, system or method listed is relevant to your study. If you are not sure if a list item applies to your research, read the appropriate section before selecting a response.

Materials & experimental systems

n/a	Involved in the study	
☒	☐	Antibodies
☒	☐	Eukaryotic cell lines
☒	☐	Palaeontology and archaeology
☐	☒	Animals and other organisms
☒	☐	Clinical data
☒	☐	Dual use research of concern
☒	☐	Plants

Methods

n/a	Involved in the study	
☒	☐	ChIP-seq
☒	☐	Flow cytometry
☒	☐	MRI-based neuroimaging

Animals and other research organisms

Policy information about studies involving animals; ARRIVE guidelines recommended for reporting animal research, and Sex and Gender in Research

Laboratory animals	90 C57BL/6 male Mice, (60 days old, ~25g; Envigo, Jerusalem, Israel), were housed together (up to 5 per cage) at 22 ± 2°C, 40-70% humidity, under 12-hour reversed light/dark cycles. Mice were allowed water and laboratory rodent chow ad libitum.
Wild animals	The study did not involve wild animals.
Reporting on sex	The study only involved male animals, as the behavioral paradigm was a modified version of prior studies (Heinz et al. 2017).
Field-collected samples	The study did not involve samples collected from the field.
Ethics oversight	All experimental procedures were approved by the University of Haifa Ethics and Animal Care Committee, and the Israeli ministry of health (Authorization numbers 515/17 and 708/20) and adequate measures were taken to minimize pain and discomfort.

Note that full information on the approval of the study protocol must also be provided in the manuscript.

Plants

Seed stocks	*Report on the source of all seed stocks or other plant material used. If applicable, state the seed stock centre and catalogue number. If plant specimens were collected from the field, describe the collection location, date and sampling procedures.*
Novel plant genotypes	*Describe the methods by which all novel plant genotypes were produced. This includes those generated by transgenic approaches, gene editing, chemical/radiation-based mutagenesis and hybridization. For transgenic lines, describe the transformation method, the number of independent lines analyzed and the generation upon which experiments were performed. For gene-edited lines, describe the editor used, the endogenous sequence targeted for editing, the targeting guide RNA sequence (if applicable) and how the editor was applied.*
Authentication	*Describe any authentication procedures for each seed stock used or novel genotype generated. Describe any experiments used to assess the effect of a mutation and, where applicable, how potential secondary effects (e.g. second site T-DNA insertions, mosiacism, off-target gene editing) were examined.*
